# Segregation of short-wavelength-sensitive (S) cone signals in the macaque dorsal lateral geniculate nucleus

**DOI:** 10.1111/j.1460-9568.2009.06939.x

**Published:** 2009-10

**Authors:** Sujata Roy, Jaikishan Jayakumar, Paul R Martin, Bogdan Dreher, Yuri B Saalmann, Daping Hu, Trichur R Vidyasagar

**Affiliations:** 1 Department of Optometry & Vision Sciences, University of MelbourneCarlton, Vic. 3053, Australia; 2 National Vision Research InstituteCarlton, Vic., Australia; 3 School of Medical Sciences & Bosch Institute, University of SydneyNSW, Australia; 4 Sydney Node of ARC Centre of Excellence in Vision Science, University of SydneySydney, NSW 2006, Australia

**Keywords:** color vision, dorsal lateral geniculate nucleus, koniocellular, macaque, short-wavelength-sensitive cones

## Abstract

An important problem in the study of the mammalian visual system is whether functionally different retinal ganglion cell types are anatomically segregated further up along the central visual pathway. It was previously demonstrated that, in a New World diurnal monkey (marmoset), the neurones carrying signals from the short-wavelength-sensitive (S) cones [blue–yellow (B/Y)-opponent cells] are predominantly located in the koniocellular layers of the dorsal lateral geniculate nucleus (LGN), whereas the red–green (R/G)-opponent cells carrying signals from the medium- and long-wavelength-sensitive cones are segregated in the parvocellular layers. Here, we used extracellular single-unit recordings followed by histological reconstruction to investigate the distribution of color-selective cells in the LGN of the macaque, an Old World diurnal monkey. Cells were classified using cone-isolating stimuli to identify their cone inputs. Our results indicate that the majority of cells carrying signals from S-cones are located either in the koniocellular layers or in the ‘koniocellular bridges’ that fully or partially span the parvocellular layers. By contrast, the R/G-opponent cells are located in the parvocellular layers. We conclude that anatomical segregation of B/Y- and R/G-opponent afferent signals for color vision is common to the LGNs of New World and Old World diurnal monkeys.

## Introduction

Afferent neuronal signals in the primate visual system are conveyed in multiple parallel channels. These channels are fed by parallel arrays of retinal ganglion cells, each with its distinct functional and morphological characteristics (for review see Dacey, 1999; Masland & Martin, 2007; Werblin & Roska, 2007). The ganglion cells that project to the parvocellular and magnocellular layers of the dorsal lateral geniculate nucleus (LGN) have been extensively studied in both Old World and New World monkeys (Wiesel & Hubel, 1966; Dreher *et al.*, 1976; Sherman *et al.*, 1976; Norden & Kaas, 1978; Schiller & Malpeli, 1978; Hicks *et al.*, 1983; Derrington & Lennie 1984; Derrington *et al.*, 1984; Yeh *et al.*, 1995; Martin *et al.*, 1997; White *et al.*, 1998; Blessing *et al.*, 2004) and in prosimian primates (Norton & Casagrande, 1982; Irvin *et al.*, 1986, 1993; Holdefer & Norton, 1995; Yamada *et al.*, 1998). Linking the anatomy and function of the third (koniocellular) subdivision of the afferent visual pathway has been more problematic. Where measured, the functional properties of koniocellular cells also show great diversity (Irvin *et al.*, 1986; Holdefer & Norton, 1995; White *et al.*, 1998; Xu *et al.*, 2001). Furthermore, in Old World monkeys the koniocellular layers are thin and are not clearly demarcated from the main parvocellular and magnocellular layers (Kaas *et al.*, 1978; Hendry & Reid, 2000). The question of whether the koniocellular layers are involved in color vision has been addressed in a New World diurnal monkey, the common marmoset. In this species the koniocellular layer K3 is sandwiched between the main magnocellular and parvocellular layers of the LGN and is broad enough (Kaas *et al.*, 1978; Spatz, 1978) to make histological reconstruction of the sites of physiological recordings relatively straightforward. These experiments showed that S-cone input cells in the marmoset LGN are segregated in koniocellular layer K3 (Martin *et al.*, 1997; White *et al.*, 1998; Solomon *et al.*, 1999; Szmajda *et al.*, 2006). In macaques, S-cone-initiated signals are known to be carried by the small bistratified ganglion cells (Dacey & Lee, 1994) and the homologous small bistratified cell in marmosets projects to layer K3 of the LGN (Szmajda *et al.*, 2008).

One early study that characterised blue–yellow (B/Y)-opponent cells in the macaque LGN (Schiller & Malpeli, 1978) localised them within the middle two of the six main layers of the LGN. However, that study, in common with several other functional studies of the macaque LGN (Wiesel & Hubel, 1966; Dreher *et al.*, 1976; Hicks *et al.*, 1983; Derrington & Lennie 1984; Derrington *et al.*, 1984), did not specifically distinguish the koniocellular layers from the parvocellular or magnocellular layers.

Here, we have investigated the properties of cells in the macaque LGN using a battery of functional tests including cone-isolating stimuli, and performed histological reconstruction of cell locations with explicit delineation of the koniocellular regions of the LGN. We also measured the latency of cell responses (action potentials) to electrical stimulation of the optic chiasm. The distributions of electrically-evoked latencies in magnocellular and parvocellular cells are non-overlapping ranges (Dreher *et al.*, 1976; Vidyasagar *et al.*, 2002), so we asked whether the koniocellular pathway in macaques can also be distinguished in this way. We have also used the responses to sine wave-modulated grating presentations to estimate the centre and surround sizes and sensitivities of the receptive fields of the red–green (R/G)- and B/Y-opponent cells. Preliminary results have been already published in the form of an abstract (Roy *et al.*, 2007).

## Materials and methods

### Anaesthesia and surgery

Electrophysiological recordings were made from four adult *Macaca fascicularis* (5–5.6 kg, two male and two female). Anaesthesia was induced with intramuscular administration of a mixture of ketamine hydrochloride (15 mg/kg, Ketamil; Parnell Laboratories, Australia Pty. Ltd.) and xylazine (2 mg/kg, Rompun; Bayer Australia Ltd.) and maintained initially by supplementary administration of ketamine and xylazine as required. Both cephalic veins were catheterised and the trachea was cannulated. A thermister was inserted under the right scapula for monitoring body temperature and the animal was placed in the stereotaxic frame with its vertebrae suspended to aid clearance of pulmonary secretions. After the induction of skeletal muscle paralysis with vecuronium (0.7 mg/kg i.v.; Norcuron, Organon), anaesthesia and muscle relaxation were maintained with an intravenous infusion of sufentanil (2–6 μg/kg/h; Sufentanil, Jaansen-Cilag) and vecuronium (0.2 mg/kg/h). The electrocardiogram and electroencephalogram were continually monitored to help in ensuring adequate depth of anaesthesia. Through the second venous line, a slow infusion of 5% glucose in normal saline was administered throughout the experiment to maintain a total fluid administration of ∼200 mL per day. The ventilation was adjusted to maintain end-tidal CO_2_ between 3.6 and 3.8%. The body temperature was kept to ∼36°C using a servocontrolled heating blanket. Experiments were approved by the Animal Ethics Committee of the University of Melbourne and conformed to NIH guidelines and the Australian code of practice for the care and use of animals for scientific purposes.

### Electrophysiological recordings and visual stimuli

A cranial opening was made between Horsley–Clark co-ordinates anterior 2–12 and lateral 7–15 mm. Small craniotomies were made near the optic chiasm (around Horsley–Clark co-ordinates anterior 19 and lateral 2 mm) for inserting stimulating electrodes. Epoxy-coated tungsten microelectrodes (impedance 5–12 MΩ; Frederick Haer Co, Bowdoinham, ME, USA) were used for the extracellular recordings from single neurons in the LGN. The pupils were dilated with atropine (1%) and the eyes fitted with gas-permeable contact lenses. The position of the optic nerve head and the fovea of each eye were plotted at regular intervals during the experiment using a Fundus camera equipped with a rear-projection device. The refractive error of each eye was determined by retinoscopy and corrected with the use of appropriate lenses. A 3-mm-diameter artificial pupil was used for each eye.

After initial hand-plotting of the receptive field in relation to the fovea, drifting achromatic (luminance modulated) sine-wave gratings were used to establish the optimal spatial and temporal frequencies and optimal direction of movement, except in cases where achromatic gratings gave poor responses. In these latter cases, the best chromatic stimulus [long-wavelength (L)–medium-wavelength (M) chromatic or S-cone-isolating; see Table 1) was used to ascertain the near-optimal spatial frequency, temporal frequency and direction of movement. The visual stimuli were generated using either a VSG Series Three or a Visage video signal generator (Cambridge Research Systems, Cambridge, UK) and presented on a Reference Calibrator Plus monitor (Barco, Kortrijk, Belgium) at a frame refresh rate of 80 Hz. The VSG system incorporates a photometric feedback system for colorimetric specification and gamma correction to allow direct specification of stimuli in Commission internationale de l'éclairage (CIE) coordinates (x, y, Y). Mean luminance was between 25 and 60 cd/m^2^. The accuracy of the system was verified with a PR-650 photometer (Photo Research, Palo Alto, CA, USA). Spatial frequency tuning functions were measured using moving sine-wave gratings of eleven different spatial frequencies (0.01, 0.1, 0.2, 0.4, 0.8, 1.6, 2.4, 3.2, 4.8, 6.4 & 12.8 cycles/°). Nine different drift frequencies (0.5, 1, 2, 4, 6, 8, 12, 16 and 32 cycles/s) were used to measure the temporal frequency tuning. The directional tuning curves were measured using drifting gratings at 22.5° steps. The gratings were presented as circular patches, 6° in diameter. Contrast sensitivity functions were derived using achromatic sine-wave gratings drifting at optimal spatial frequency and orientation, at nine different contrasts (1.56, 3.12, 6.75, 9.35, 12.5, 25, 37.5, 50 and 100%). Measurements were normally made at 4 Hz temporal frequency in order to reduce the confounding effects of centre–surround latency differences.

**Table 1:** Cone contrasts of the short-wavelength (S), medium-wavelength (M) and long- wavelength (L) respectively for the various stimuli used in this study

	S (423 nm)	M (530 nm)	L (558 nm)
S-cone-isolating	0.4621	0.0095	0.0013
Silent L-cone	−0.1688	−0.4736	−0.0554
Silent M-cone	0.3156	−0.0176	0.415
Achromatic	1	1	1
LM chromatic	0.078	−0.2601	0.1907

Spectral absorbance templates (nomograms) with peak wavelengths at 560, 530 and 430 nm were generated using a polynomial template (Lamb, 1995). Lens absorbance was corrected using published values for human lens (Wyszecki & Stiles, 1982). The effect of receptor self-screening was estimated assuming axial absorbance of 1.5% and outer segment length 20 μm. No correction for macular pigment was made. The cone contrast for a given stimulus was calculated for each nomogram by convolution with the [x, y, Y] coordinates of the grating components via the Judd–Voss modified CIE 1931 color matching functions (Brainard, 1997).

The S-cone-isolating gratings were modulated between CIE co-ordinates (0.294, 0.268) and (0.336, 0.414) through the grey point (0.317, 0.335). The M-cone- and L- cone-selective gratings were generated using the red and green monitor phosphors. Table 1 shows the cone contrast for the visual stimuli that we used. These stimuli enabled us to characterise the cone inputs to the cells and the presence of any color opponency in the LGN cells. The reader should note that the presence of S-cone contrast in the M- and L-cone-selective stimuli is not a major issue, as other studies (Sun *et al.*, 2006a,b;) and our own tests showed that all parvocellular and magnocellular cells showed little or no evidence of input from S-cones. Peristimulus time histograms (PSTHs) were built from the unit response to 2–4 s of the grating drifting at the optimum temporal frequency and repeated 3–5 times. The amplitude of the first harmonic component of the fast Fourier transform (FFT) of the PSTH was extracted to provide a measure of the cell’s response. A difference-of-Gaussians (DOG) model (Rodieck, 1965) was applied to the spatial frequency response function to calculate the radius of the receptive field centre and the centre and surround sensitivities (Croner & Kaplan, 1995; Solomon *et al.*, 2002).

In two monkeys, we inserted a pair of concentric stimulating electrodes (NEX-100; Clark Electromedical Instruments, Reading, UK), one on either side of the optic chiasm, and measured the latencies of evoked action potentials (spikes) in the LGN to both ipsilateral and contralateral electrical stimulation (constant current pulses of 0.5–10 mA and duration 100–200 μs at 0.25–1 Hz).

### Histology

**Fig 1:**
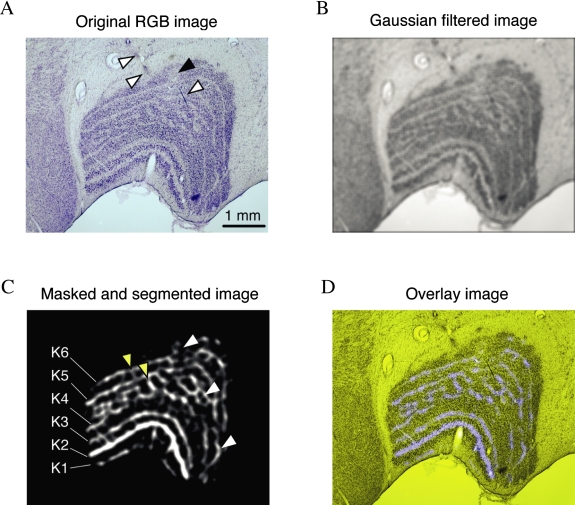
Sequence of digital image processes carried out on (A) a Nissl-stained section to define the koniocellular layers and their ‘bridges’ within the LGN. (B) After applying a Gaussian filter to remove the higher spatial frequencies, (C) the low-frequency regions are segmented using an ‘opening’ filter. (D) This image is superimposed as the blue channel of the original image. The arrowheads in A indicate the paths of two electrode tracks. The black filled arrowhead indicates the site of an electrolytic lesion. The small yellow arrowheads in C indicate two koniocellular ‘bridges’ spanning the external parvocellular layers and the large white arrowheads show the positions of three B/Y cells encountered on the electrode path indicated in A.

In each penetration one to three electrolytic lesions (6 μA for 6 s, electrode negative) were made. At the end of the experiment, the macaque was given a lethal intravenous dose of pentobarbitone sodium (5 mL of Nembutal, 60 mg/mL; Merial Australia Pty Ltd) and transcardially perfused with 0.1 m phosphate-buffered saline (PBS), followed by 4% paraformaldehyde in 0.1 m PBS. The brain was removed and sunk in 30% sucrose in 0.1 m phosphate buffer. Alternate frozen coronal 50-μm sections through the LGN were stained with Cresyl violet for Nissl substance. A Zeiss Axiocam digital camera was used to make digital micrographs and the stacks of images were analysed using custom software written in Matlab (Image Processing Toolbox; Mathworks, Natick, MA, USA). Each electrode track with its physiologically characterised cells was reconstructed with the help of the electrolytic lesions, microdrive readings and changes of eye laminae. All physiologically identified cells were allocated their respective laminar locations. Identification of the koniocellular zone, including the ‘bridges’ spanning parvocellular layers, was assisted by spatial low-pass filtering of the images using an image manipulation software (Adobe Photoshop or Matlab). Such low-pass spatial filtering (roughly equivalent to optical blurring) reduces the spatial contrast of the koniocellular layers, because the cell bodies in these layers are relatively small and their density is relatively low (LeGros Clark, 1941; von Noorden & Middleditch, 1975; Hendry, 1992; Ahmad & Spear, 1993). The effectiveness of this simple procedure is evident when one inspects Fig. 1. Low-pass-filtered regions can be objectively segmented using standard Matlab image processing toolbox functions (*imfilter, imopen*; Mathworks). The borders of the koniocellular layers and their extensions along with the main parvocellular and magnocellular layers were outlined by inspection and the positions of labelled cells were reconstructed. The arrowheads in panel A in Fig. 1 indicate the paths of two electrode tracks in this section. The black filled arrowhead indicates the site of an electrolytic lesion. The yellow arrowheads in panel C indicate two koniocellular ‘bridges’ spanning the external parvocellular layers. The white arrowheads show the positions of three B/Y cells (the terms ‘B/Y cells’ and ‘B/Y-opponent cells’ are used interchangeably; similarly with ‘R/G’) encountered on the electrode path indicated in panel A.

## Results

### LGN cells with S-cone inputs

**Fig 2:**
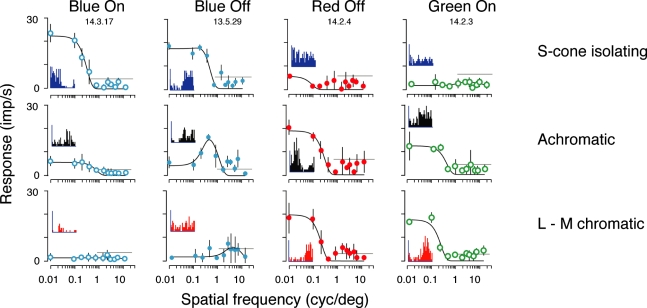
Classification of LGN cells. Each column shows spatial frequency transfer functions for one cell of the indicated response type. Each row shows responses to one stimulus type. The top row shows responses to stimuli modulating only the S-cones and the second row shows responses to achromatic gratings modulating all three cone types in additive phase. The lower row shows responses to R/G (L–M) chromatic modulation. The temporal frequency of the drift was 4 Hz and the orientation was optimised for each cell. The cone contrasts for each stimulus are shown in Table 1. The insets show PSTHs made with a bin width of 10 ms from the response to a low (0.01 cycles/deg) spatial frequency condition in each panel. The phase of Blue On cell responses to achromatic stimuli could be either On or Off and, in this cell, it is Off. The abscissa is 0.25 s and the ordinate 10 impulses/s. The horizontal grey lines show the amplitude of the f1 component of the FFT in the absence of spatial contrast.

We recorded single-unit activity of 92 cells. The receptive fields of these cells were located between 2 and 12 degrees from the centre of the fovea. Most of the electrode tracks did not extend into the magnocellular layers, either because the line of penetration was outside the magnocellular layers or because we intentionally stopped when the physiological recordings indicated that the electrode had entered the magnocellular layers. Based upon the responses to the cone-modulating stimuli, we could identify each cell as a R/G cell (i.e., primarily antagonistic L-cone and M-cone inputs), a B/Y cell (i.e., with S-cone inputs) or an achromatic cell (i.e., primarily with in-phase L-cone and M-cone inputs). We report here mainly the results related to the R/G and B/Y cell classes, which made up 88 of the 92 recorded cells. Figure 2 shows the responses of four cells (a Blue On, a Blue Off, a Red Off and a Green On) to drifting sine-wave gratings modulating the cones in three different ways. The top row shows spatial frequency response functions of the first harmonic response to S-cone modulation, the middle row shows the responses to achromatic gratings modulating all three cone types in-phase and the lower row shows responses to L-M chromatic modulation. The two B/Y cells in the first two columns show vigorous responses to the S-cone modulation and weak responses to achromatic and L-M chromatic gratings. In contrast, the two cells whose responses are illustrated on the right show no response to S-cone-selective gratings but vigorous responses to L-M chromatic and to achromatic stimuli. These two cells were identified as typical parvocellular R/G-opponent cells, as the cone contrast in the L-M modulation stimulus was 16% of that for the achromatic stimuli, yet the response to the isoluminant chromatic stimuli was at least as vigorous as the response to achromatic contrast.

**Fig 3:**
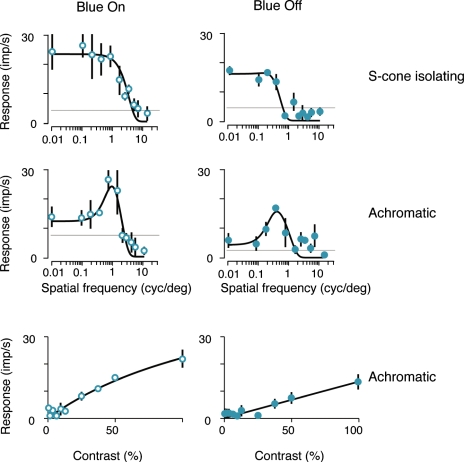
Contrast sensitivity functions for a Blue On cell and a Blue Off cell (same cell as in Fig. 2) for achromatic gratings (bottom row) along with their responses at different spatial frequencies for S-cone-isolating and achromatic gratings (top two rows). The drift frequency used was 4 Hz for both cells. The contrast sensitivity functions were done at the optimal spatial frequency and direction of movement for each cell. The grey horizontal lines show the amplitude of the f1 component of the FFT in the absence of spatial contrast.

B/Y cells often respond to achromatic stimuli (Fig. 3). However, responses to S-cone-isolating stimuli were always more vigorous than responses to achromatic stimuli. Contrast sensitivity functions of both a Blue On cell (Fig. 3, bottom left) and a Blue Off cell (Fig. 3, bottom right) obtained with achromatic gratings with a spatial frequency evoking optimal responses with S-cone-isolating stimuli show that only at very high luminance contrasts do the B/Y cells show any response approaching that seen with S-cone-isolating stimuli.

Background, or maintained, activity (f0 component of the FFT) was measured at the mean screen luminance in the absence of spatial contrast. The mean ± SD background discharge rate of R/G cells (13.19 ± 7.74; *n* = 47) was not significantly different from that of B/Y cells (11.96 ± 11.83; *n* = 18, *P* = 0.14, Wilcoxon rank-sum test).

No cells in our sample showed overt direction selectivity. However, as orientation sensitivity has been reported both for retinal ganglion (Levick & Thibos, 1982; Passaglia *et al.*, 2002) and LGN (Vidyasagar & Urbas, 1982; Shou & Leventhal, 1989; Smith *et al.*, 1990; Xu *et al.*, 2002; Tailby *et al.*, 2008) cells, we characterised orientation selectivity with an orientation index metric, which varies between zero (no orientation selectivity) and unity (Levick & Thibos, 1982; Forte *et al.*, 2005). On average both B/Y and R/G populations showed mild orientation selectivity for achromatic gratings (mean orientation index for B/Y cells, 0.144 ± 0.130, *n* = 9; mean for R/G cells, 0.105 ± 0.081, *n* = 29; *P* = 0.81, Wilcoxon paired rank-sum test). A more detailed study of orientation bias and its spatial frequency dependence was not made.

### Size and sensitivity of receptive field centre and surround

**Fig 4:**
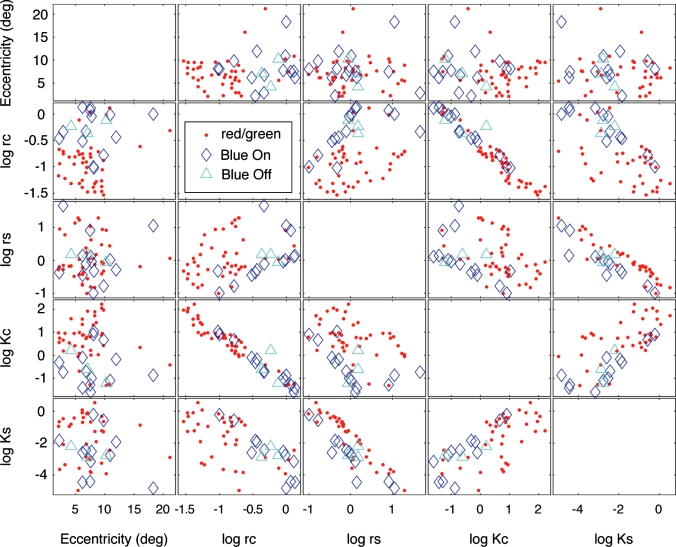
Relationships between centre radius (rc), surround radius (rs), centre sensitivity (Kc), surround sensitivity (Ks) and visual field eccentricity for R/G and B/Y cells. Radii are in degrees and sensitivity in impulses/s/deg^2^.

Using a DOG model (Rodieck, 1965) we estimated the radii and sensitivity of receptive field centre and surround from the response amplitudes obtained with achromatic sine-wave gratings of optimal spatial and temporal frequencies and orientation (Croner & Kaplan, 1995; White *et al.*, 2001). The matrix shown in Fig. 4 plots centre and surround radii, their respective sensitivities and the visual field eccentricity against each other for each of the 36 R/G-opponent cells and 16 B/Y-opponent cells for which complete data are available. The R/G-opponent cells have, on average, smaller centre radii than B/Y-opponent cells (Wilcoxon ranked-sum test, *P* < 0.01). This has been noted earlier for both macaques (Wiesel & Hubel, 1966; Schiller & Malpeli, 1978; Solomon *et al.*, 2005) and marmosets (Tailby *et al.*, 2008). The average centre sizes of both cell types show little change within 10° of eccentricity, from where most of our sample comes. Centre sensitivity was also inversely proportional to the radius with a regression slope close to −2. For R/G-opponent cells, *K*_c_ = 0.08 and *r*_c_^−2.04^ (*r*^2^ = 0.94, *P* < 0.01) and for B/Y-opponent cells, *K*_c_ = 0.07 and *r*_c_^−2.14^ (*r*^2^ = 0.89, *P* < 0.01), where *K*_c_ is centre sensitivity and *r*_c_ is centre radius. That the slope of the regression is close to −2 indicates that the centre sensitivity is inversely proportional to the square of the radius.

For a subset of B/Y cells (*n* = 14) we measured spatial frequency tuning for achromatic and S-cone-isolating gratings. Four of these cells did not respond at > 5 impulses/s to any achromatic grating presented. For the remaining cells we fitted the responses to a DOG model as described above. Centre radius for achromatic gratings (0.529 ± 0.451, *n* = 10) was not significantly different from the centre radius for S-cone-isolating gratings (mean 0.529 ± 0.451, *n* = 10; *P* = 0.92, Wilcoxon paired rank-sum test). The centre radii measured for achromatic and S-cone-isolating gratings were mildly correlated (correlation coefficient 0.55, *P* = 0.10). Consistent with results obtained from B/Y-opponent cells in marmoset LGN (Tailby *et al.*, 2008), we did observe wide variation in the shape of the achromatic spatial tuning function. We further characterized the spatial tuning of B/Y cells using the low-cut ratio statistic (Tailby *et al.*, 2008). The low-cut ratio is calculated as the response to the lowest frequency divided by that to the best frequency. The low-cut ratio can vary between 0 (indicating that responses are completely attenuated at low frequencies) and 1 (indicating that the tuning function is low pass). The B/Y cell responses to S-cone gratings was more low-pass (mean ± SD low-cut ratio 0.998 ± 0.005, *n* = 10) than the response to achromatic gratings (0.754 ± 0.332, *n* = 10) but the difference was not significant (*P* = 0.12, Wilcoxon paired rank-sum test). We did not undertake a more detailed analysis of achromatic spatial tuning in B/Y cells.

### Laminar location of color-opponent LGN cells

**Fig 5:**
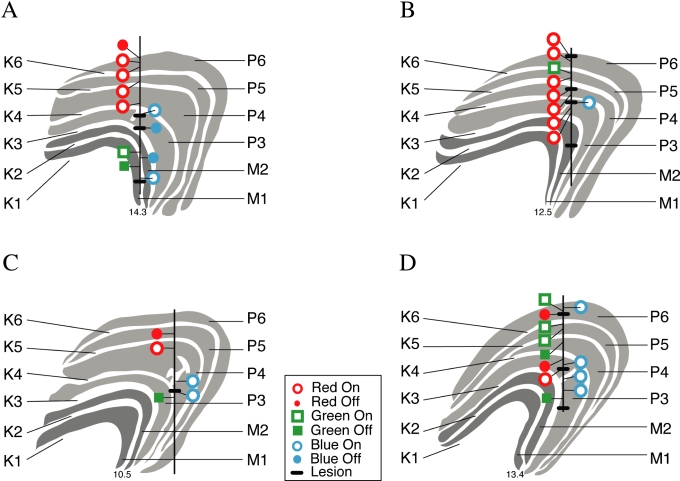
Electrode tracks, one from each of the four animals, with locations of the functionally identified cells, reconstructed from three or four Nissl-stained sections. The koniocellular extensions into the parvocellular layers (into P3 in A, B and D and P4 in C) near the electrode tracks are shown, but not all such koniocellular bridges in the sections are shown in the figure. The inset provides the key for cell types. The horizontal black lines on the electrode tracks indicate the sites of electrolytic lesions.

**Fig 6:**
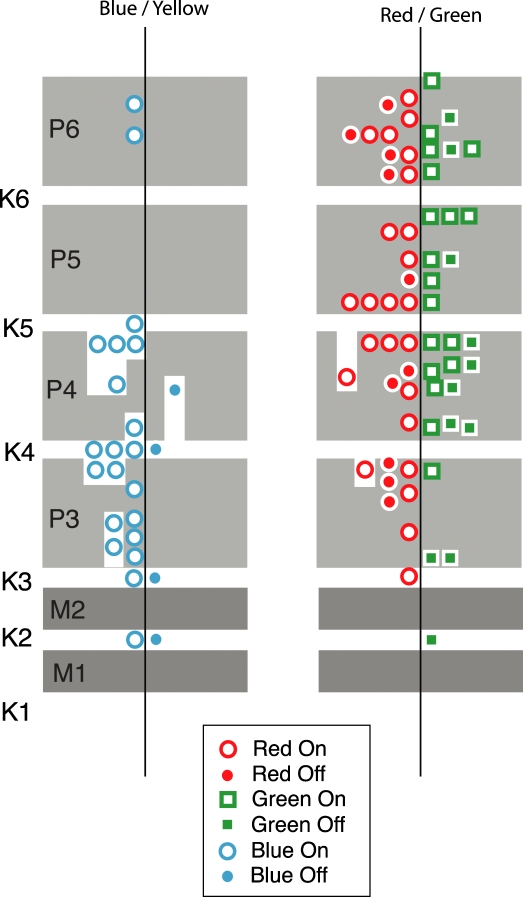
Pooled data of the laminar distribution of cell types within the LGN from all four monkeys (*n*=88). The cells are placed along the schematised depth of the LGN, roughly proportionate to their distance from the immediately ventral koniocellular layer. In the left panel, the B/Y cells are shown with Blue On cells left of the vertical line and Blue Off cells to the right. In the right panel, R/G cells are shown with Red On and Red Off left of the vertical line and Green On and Green Off to the right. Where cells were localised in the koniocellular bridges in the parvocellular layers, such bridges are shown in the figure along with the cells localised within them. All except six cells (three R/G-opponent and three B/Y-opponent) were localized in the expected eye-specific layer.

The locations of physiologically identified cells were located in histological sections as explained in Materials and Methods. The low-pass filtering (or just optical blurring) delineated the koniocellular extensions and bridges intruding into the parvocellular layers. Such intrusions were more common in the inner parvocellular layers, P3 and P4, and were most prominent in P4 (see Fig. 1D). Figure 5 shows four reconstructed electrode tracks in the LGN (one track from each of the four animals). In each case the B/Y-opponent cells were located in or close to the koniocellular regions identified histologically. The distribution of color-opponent cells from 15 electrode tracks in four monkeys are pooled and shown distributed across the geniculate laminae in Fig. 6. They are identified by their laminar position, whether they were On or Off and whether they were R/G- or B/Y-opponent, and placed at the relative distance from the intercalated layers. The figure also distinguishes cells that were located in koniocellular intrusions and bridges in the parvocellular laminae. We have categorised these cells as being within the koniocellular subdivision of the LGN. We restricted our analysis to cells that were recorded dorsal to the magnocellular layers. Of the 88 color-opponent cells shown in Fig. 6, four cells were excluded as they were in the koniocellular layer K2 between the two magnocellular layers. The distribution of the main cohort of the remaining 84 color-opponent cells across the laminae was analysed with reference to the cell type, i.e., whether they had On or Off centre receptive fields and whether they were R/G- or B/Y-opponent.

There were 61 cells that received antagonistic inputs from M- and L-cones and 23 cells that received inputs from S-cones. Forty-two of the R/G-opponent cells and 19 of the B/Y-opponent cells were of the On-centre type. Overall, contrary to report of Schiller & Malpeli (1978), we did not find any indication of segregation of On- and Off-centre cells to the outer (K5, P5, K6, P6) and middle (K3, P3, K4, P4) laminae respectively (χ^2^ = 0.505, *P* = 0.48). When the R/G cells alone were analysed, a mild tendency for such segregation was seen (χ^2^ = 2.16, *P* = 0.14). Sixty-three per cent (12 out of 19) of Off-centre cells and 43% (18 out of 42) of the On-centre cells were located in the middle laminae.

The most clear-cut segregation found in our sample of geniculate cells (see Fig. 6) was with regard to the distribution of R/G-opponent cells vs. cells with S-cone inputs. They differed in two ways. First, the B/Y-opponent cells were found mostly within the middle laminae (K3–P4) rather than in the outer ones (K5–P6). While R/G-opponent cells were nearly equally common in the middle and outer layers (30 of the 61 cells being in the middle layers), most of the B/Y-opponent cells (20/23) were found in the middle layers (χ^2^ = 9.89, *P* = 0.002). Second, the B/Y cells tended to be located in the koniocellular regions of the LGN. As mentioned in Materials and Methods, the koniocellular bridges and extensions into the parvocellular layers were also included in what we term the ‘koniocellular region’ of the LGN. When thus analysed, 74% of the B/Y cells (17/23) were located in such koniocellular regions, whereas only 5% (3/61) of the R/G-opponent cells were located in these same regions (χ^2^ = 43.8, *P* < 0.001). This is a high degree of segregation, especially considering the errors in estimation that can be potentially introduced due to the thinness of the koniocellular layers. The reader should note that the histological reconstructions were all done ‘double-blind’, i.e., cell locations were identified without knowledge of their physiological classification.

### Latency of the responses of LGN neurons to electrical stimulation of their retinal afferents

**Fig 7:**
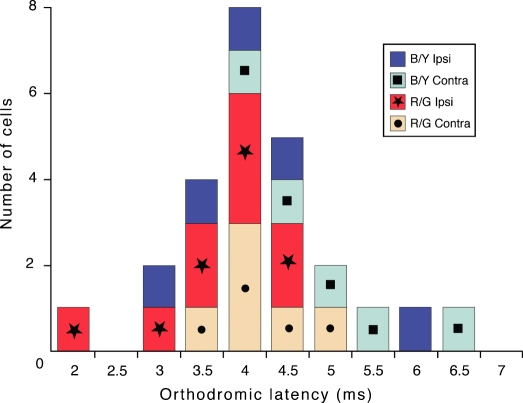
Distribution of latencies of R/G-opponent and B/Y-opponent LGN cells to electrical stimulation from electrodes placed to straddle the optic chiasm. The inset provides the key to cell type (R/G- or B/Y-opponent) and latency to electrical stimulation of either ipsilateral or contralateral optic chiasm.

In two monkeys we obtained orthodromic spike-response latencies to electrical stimulation of the optic chiasm. There was a wide variation in the orthodromic latencies for both R/G- and B/Y-opponent cell type (Fig. 7). It was not always possible to evoke orthodromic action potentials in the LGN cells with the current range we used and not all the LGN cells that could be driven by electrical stimulation were driven from both sides of the chiasm. The mean latency for contralaterally-evoked responses of B/Y-opponent cells was only slightly longer (5.22 ± 0.19 ms, mean ± SEM; *n =* 5) than the mean latency for R/G-opponent cells (4.4 ± 0.09 ms, *n* = 6) and this difference was not statistically significant (Mann–Whitney *U*-test, *P* = 0.23). Similarly, only a weak trend was seen for ipsilaterally-evoked spike-response latencies (4.38 ± 0.22 ms, *n* = 5, vs. 3.9 ± 0.9 ms, *n* = 9; Mann–Whitney *U*-test, *P* = 0.63). In summary, although the longest-latency cells were B/Y cells, there was extensive overlap between the B/Y- and R/G-opponent cells. Therefore, we conclude that that the latencies of responses of LGN cells to electrical stimulation of the optic chiasma cannot be taken as a definitive criterion for identification of LGN cells as koniocellular or parvocellular.

## Discussion

Our results suggest that the distribution of color-selective cells within the macaque’s LGN follows a pattern similar to that described in the marmosets (Martin *et al.*, 1997; Szmajda *et al.*, 2006). The fact that the koniocellular layers of the macaque LGN are very thin and dispersed has hampered study of the functional properties of koniocellular regions in this species. Despite the noise that this anatomical ‘inconvenience’ introduces into the data, we find a clear segregation of cells with S-cone inputs (B/Y-opponent cells) to the koniocellular regions of the macaque’s LGN. It is to be noted that we have included the koniocellular bridges within the parvocellular layers as part of our target, koniocellular, region. About 40% (9/23) of the B/Y cells were in such koniocellular extensions.

Our study has not systematically included the koniocellular layers deeper to the two magnocellular layers (K1 and K2) and any of their extensions into neighboring magnocellular regions. However, it is interesting that the only two B/Y cells recorded ventral to K3 were both found in the koniocellular layer, K2. It will be worthwhile to explore whether, similar to the dorsal layers, the ventral layers of the LGN contain koniocellular regions within them which are the targets of S-cone signals.

We found that the latency of the spike responses to electrical stimulation of the optic chiasma cannot be used as a definitive criterion to identify koniocellular inputs to the macaque LGN. Even with our limited sample, there was considerable overlap between the conduction velocities of axons of R/G-opponent and B/Y-opponent ganglion cells. A similar result was obtained by Solomon *et al.* (2005) who found that R/G and B/Y ganglion cells in intraocular recordings showed substantial overlap in antidromically-evoked latencies. By contrast, Irvin *et al.* (1986) found slow conduction velocity afferents to the interlaminar–koniocellular layers of the LGN in the nocturnal prosimian primate *Galago.* As this species lacks a functional S-cone pathway (Deegan & Jacobs, 1996), it can be speculated that axons of ‘non-blue’ koniocellular afferents have slower conduction velocity, but our sample had too few non-blue koniocellular cells to address this question. It is also worth noting that, if the assessment of conduction velocities of retinal axons is based on measurements over longer distances than those from the optic chiasm, LGN response latencies might be more useful in identification of very slowly conducting vs. slowly conducting retinal afferents, as has indeed been shown for the functional identification of retinogeniculate afferents in the cat (Cleland *et al.*, 1976).

The inverse relationship between the radii of the receptive field centre and centre sensitivity with a regression slope of -2 for all cells indicates that, as in New World diurnal monkeys (White *et al.*, 2001), the integrated responsivity of the receptive field centre is constant irrespective of the centre size. Recently, Tailby *et al.* (2008) showed that in marmosets the integrated sensitivity of Blue On cells (for S-cone-selective gratings) is higher than the integrated sensitivity of parvocellular cells (for achromatic gratings). A larger data set in the macaque may be able to show a similar difference, unless there is a genuine species variation.

Our anatomical analysis assumed that the parvocellular layers of the macaque include many extensions of the koniocellular regions, as hinted earlier on the basis of the neurochemical identification of LGN cells (Hendry & Yoshioka, 1994). We found that simple low-pass spatial filtering of Nissl-stained sections can enhance the visibility of not only the main koniocellular layers but also the koniocellular extensions into the parvocellular layers. We found that, with regard to the cone inputs, when these extensions are included as part of the koniocellular subdivision of the LGN they encompass the majority of the S-cone input cells. Nevertheless, further test of our assumption will require new experiments correlating the distribution of neurochemical markers for the koniocellular layers (see for review Hendry & Reid, 2000) with the pale regions revealed by spatial filtering. Our study was largely focused on color-opponent cells in the macaque LGN, and we did not make detailed analysis of cells showing ‘non-standard’ properties. Thus, our results do not preclude the possibility that the koniocellular regions of the macaque LGN may be a heteregenous population with achromatic cells intermingled with the B/Y cells.

Our study provides the most direct evidence to date in support of earlier studies (Hendry & Reid, 2000; Chatterjee & Callaway, 2003) suggesting that in macaques, as in New World monkeys, S-cone signals are carried by the koniocellular pathway. Although there is physiological evidence that many striate cortical cells with S-cone inputs may also receive magnocellular inputs (Vidyasagar *et al.*, 2002), the separation of the B/Y-opponent and R/G-opponent cells in the LGN may have a number of functional implications. First, it provides a site for selective modulation of the B/Y pathway by extraretinal inputs, both cortical and subcortical (Casagrande *et al.*, 2005; Schütz *et al.*, 2008). Second, the segregation could enable the direct koniocellular projection to the middle temporal (MT) area (Sincich *et al.*, 2004) to carry an S-cone signal to the dorsal cortical stream. There is human psychophysical evidence not only for the presence of such S-cone inputs to area MT (Morand *et al.*, 2000; but see Riecanský*et al.*, 2005) but also for the participation of the B/Y-opponent system in directing spatial attention to a greater extent than the R/G-opponent system (Li *et al.*, 2007). Demonstration of the segregation of the B/Y system in the Old World monkeys has also implications for studying possible differential influence of the two opponent pathways in various psychophysical phenomena and in clinical conditions such as blindsight (Weiskrantz, 2004).

Our findings support the suggestion that in all diurnal primates the chromatic channels are segregated between the koniocellular and parvocellular regions of the LGN. This means that in those primates which are dichromatic and in the forebears of all primates which were likely to have been dichromatic (Nathans, 1999), the koniocellular cells may be the only neurons that convey opponent color signals to the cortex. Thus the koniocellular pathway may be the primordial chromatic pathway in the mammalian lineage leading to trichromatic primates.

## Abbreviations

B/Y: blue–yellow
CIE: Commission internationale de l'éclairage
DOG: difference-of-Gaussians
FFT: fast Fourier transform
LGN: dorsal lateral geniculate nucleus
PSTH: peristimulus time histogram
R/G: red–green
